# Orbital Epidermoid Cysts: A Diagnosis to Consider

**DOI:** 10.1155/2014/508425

**Published:** 2014-09-08

**Authors:** Rania A. Ahmed, Rasha M. Eltanamly

**Affiliations:** Ophthalmology Department, Kasr Al Ainy Medical School, Cairo University, Cairo, Egypt

## Abstract

*Background*. Orbital epidermoids form a rare pathological entity that is separate from dermoid cysts. They have variable clinical and radiological presentations and they should be considered in the differential diagnosis of orbital cystic lesions. This work describes the various clinical and radiological presentations of 17 cases of epidermoid cysts and the surgical outcome. *Method*. A prospective interventional study was conducted on 17 patients diagnosed with epidermoid cysts. Patients' symptoms and signs were recorded; CT scan was done for all patients. All lesions were removed through anterior orbitotomy and histopathological diagnosis confirmed. *Results*. Mean age of patients was 16.3 years ±  10.54. Main complaints were lid swelling, masses, ocular dissimilarity, chronic pain, and ocular protrusion. Clinical signs varied from lid swelling and masses in all cases to proptosis, globe displacement, limitation of ocular motility, and scars. Radiological findings ranged from homogenous hypodense masses (58.8%) to homogenous radiolucent (17.6%) and heterogenous masses (23.5%). No recurrences following surgeries were reported throughout the follow-up (mean 18.8 months ±  0.72). *Conclusion*. Deep orbital epidemoid cysts are a separate entity that can behave like deep orbital epidermoid; however, they usually present at a relatively older age. They can be associated with increased orbital volume but not necessarily related to bony sutures.

## 1. Introduction

Epidermoid cysts are rare lesions that constitute approximately 1–1.5% of all intracranial neoplasms [1]. They can be either primary (congenital) or secondary cysts. Primary lesions are choristomas that involve displacement of epithelial elements during closure of the neural groove or other epithelial fusion lines between the third and the fifth weeks of gestation [1, 2]. Secondary epidermoid cysts result from posttraumatic implantation of surface epithelium. Those located in the orbit may arise from cutaneous, conjunctival, respiratory, or lacrimal gland epithelium. Simple epithelial cysts can occur at any age but are more commonly seen in younger adults.

Epidermoid cysts represent a distinct entity from the more common dermoid cysts although usually grouped together. Histologically, both are lined by squamous epithelium with desquamated layers of keratin; however, dermoid cysts are characterized by the presence of mesodermal elements such as hair follicles or sebaceous glands [2].

Whether dermoid or epidermoid, these cysts can be either superficial or deep. Superficial cysts are more common, simple to treat, and associated with less complication [3, 4]. Intraorbital dermoid cysts are very rare pathologies [5], representing 5–10% of all orbital dermoid cyst cases, while intraorbital epidermoids are even more infrequent [6].

Deep cysts are slowly growing masses which enlarge during lifetime. They can form through any bony suture of the orbit, including its roof. They can break through the orbital walls into the temporal cavity, sinuses, or even intracranial cavity [7]. They present in a variety of ways depending upon the suture of origin, size, rate of growth, and correlation to adjacent structures [8].

The aim of this work is to highlight epidermoid cysts as a differential diagnosis in cases of deep orbital cystic lesions, showing various clinical and radiological presentations as well as the surgical outcomes after their removal.

## 2. Materials and Methods

This is a prospective interventional case series that comprised 17 patients suffering from intraorbital epidermoid cysts from February, 2007, till May, 2012. Symptomatic patients who presented with cystic lesions on clinical examination with none delineated posterior border and placed deep to the orbital septum as well as those who presented with proptosis and intraorbital cysts on CT imaging were enrolled for the study and were scheduled for surgical removal. Inclusion in the current study was based on the histopathological analysis of the surgically excised suspected lesions. Cases with history of trauma as well as cases which were proven to be dermoids were excluded.

Detailed history was obtained from the patients or their guardians about the onset of the condition, duration, and associated symptoms. Various complaints in patients' own words were recorded. Any history of trauma or related surgical intervention was also elaborated and documented.

All included patients were thoroughly examined for overall appearance, lid and globe position, pupillary reaction, and ocular balance and motility in the cardinal directions of gaze. Visual acuity was measured and best corrected visual acuity was documented in decimal system. Proptosis, when present, was measured by Hertel exophthalmometer and any palpable masses were evaluated for their site, consistency, and local effect. Dilated fundus examination was performed and visual field was planned if there was any sign suggesting optic nerve affection. Patients were photographed and orbital CT scan with contrast was requested (coronal, axial, and sagittal cuts) and studied.

An informed consent was obtained from all patients or their guardians for surgical intervention. All procedures were performed under hypotensive general anaesthesia via anterior orbitotomy approach; however, the incision placement and design varied according to the site and accessibility of the cyst. Surgical microscope was used in cases that were showing through the conjunctiva in order to insure precise dissection and total cyst wall removal; otherwise, the surgical loupe was used. Intraoperative findings as well as any extra, unplanned steps were documented.

In cases where both the cyst wall and the bone were thin and firmly adherent, scraping, hydrogen peroxide wetted swabs, and cauterization were used to ensure destruction of the remaining lining cells.

All patients received postoperative combined antibiotic/steroid treatment topically and systemically for one week. Patients were followed up for the recurrence and sequalae of mass removal. Approval of the institutional review board was obtained, and the study and data collection conformed to all local laws and complied with the principles of the Declaration of Helsinki.

Data was collected and analyzed where descriptive statistics were calculated and the numerical data were summarized as mean and standard deviation (±SD), while categorical data were summarized in tables and percentages (%).

## 3. Results 

This study included 17 patients (10 females and 7 males) with mean age 16.3 years ±10.54 (range 5–38 y). Their complaints varied among lid swelling, chronic pain, visible mass, dissimilarity between both eyes, and rarely ocular protrusion. The frequency of different complaints as stated in patients' own words is summarized in Table 1.

Clinical examination revealed one or more of the signs shown in Table 1. Lid swelling was found in all cases, yet 9 patients showed a cystic extension to the fornix with undefined posterior border. Globe displacement, proptosis, and limitation of ocular motility were also detected; however, proptosis was mild and ranged from 2 to 4 mm (mean 2.6 mm ± 0.9).

Limitation of the ocular motility was minimal and occurred in the direction of the mass. None of the included patients had diplopia in primary gaze. Four of the included patients had a scar of previous surgery for a lesion diagnosed histopathologically as epidermoid cyst in three of them. The mean lapse between the previous surgical intervention and the current presentation was 13 months (±4.16).

Best corrected visual acuity varied from 0.6 to 1 and none of the included patients suffered from optic nerve compression.

**Figure 1:**
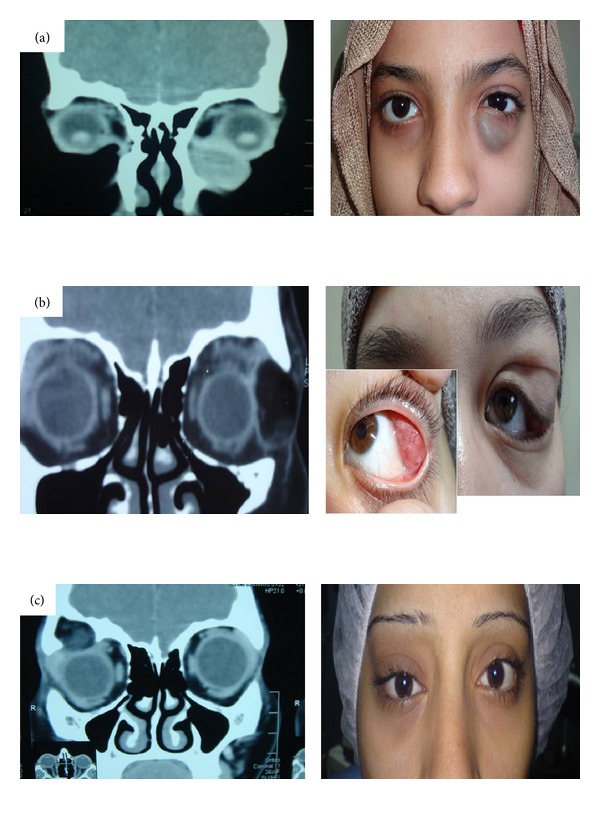
Different radiological findings (left) and correlation to clinical presentation; (a) Lt inferior nasal mass which shows a well circumscribed homogenous cyst. (b) Lt S-shaped deformity due to a radiolucent cystic swelling pushing the lacrimal gland (small caption shows the palpebral part). (c) Rt superior mass presenting with lid fullness and globe displacement while the CT shows an irregular cyst with heterogeneous appearance, bony excavation, and sclerotic edges consistent with intradiploic cyst.

**Figure 2:**
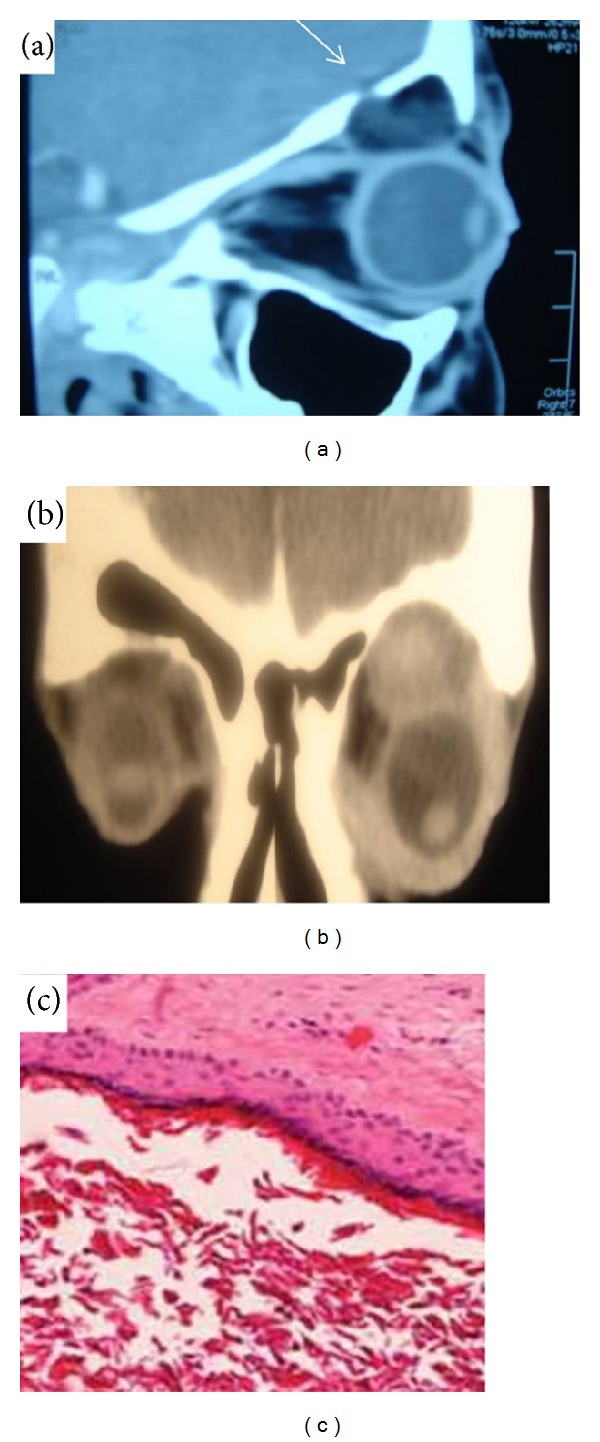
(a) Sagittal section showing a bony defect (arrow) in the orbital roof related to superior orbital cyst. (b) Superior homogenous cyst with widened orbital dimensions with obliteration of the frontal sinus. (c) Haematoxylin and eosin slide showing squamous epithelial lining with lack of skin appendages and keratin filling and chronic inflammatory cells in the wall.

Findings detected in orbital CT scans are summarized in Table 2 where the presence of an orbital cyst was the common feature in all cases. The mean length in the longest diameter was 6 cm (±0.7) with various radiodensities (Figure 1). The borders were smooth in 13 patients, yet the remaining four showed irregular borders with sclerotic edges, two of whom had an additional bony defect in the orbital roof (Figure 2(a)). Increased orbital dimensions as well as globe displacement were also detected (Figure 2(b)).

Anterior orbitotomy was the used approach for the excision of theses cysts, yet incision placement varied according to their sites in order to provide the best exposure. Transcutaneous transcrease incision was chosen to treat superior orbital cysts that were deeply seated (6 cases/35.29%) while transconjunctival approach was reserved for inferior orbital cysts (4 cases/23.53%) as well as the remaining cases presenting with visible masses through the conjunctiva (7 cases/41.17%).

All patients showed intralesional thick fluid that was grayish white in 16 cases, while it was yellowish with foul odour in one case denoting chronic abscess. Preserving the cyst intact during excision was not feasible in all cases where small defects developed during dissection; however, walls were traced and the cysts were completely excised. Specimens were thoroughly examined by two different pathologists. All cases showed epithelial lining of the cysts, filled with keratin with lack of skin appendages (Figure 2(c)).

**Figure 3:**
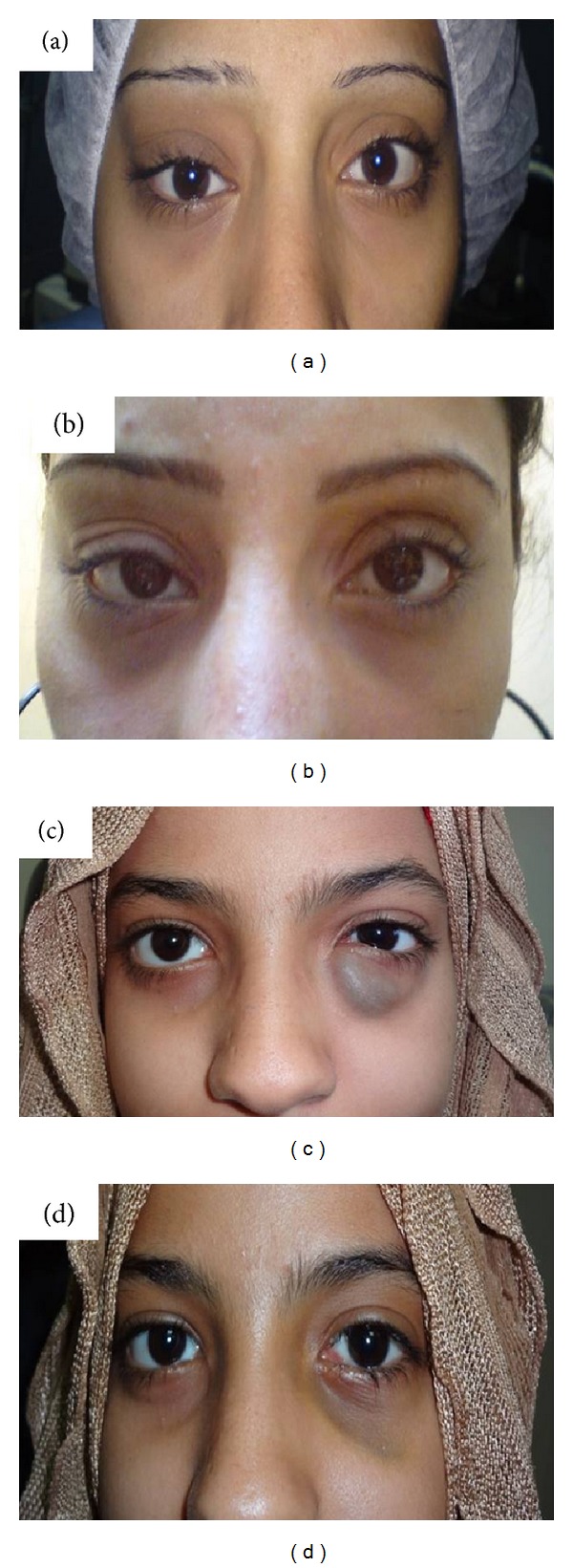
(a) Rt lid fullness, globe displacement, and proptosis in a 22-year-old female. (b) Postoperative improvement of the proptosis with residual globe displacement. (c) Lt recurrent swelling of the lower lid in a 17-year-old female. (d) Postoperative Lt enophthalmos and exaggerated tear trough detected one month after surgery.

Immediate postoperative period was uneventful and, over the follow-up period (mean 18.8 months ± 0.72), no recurrences were reported. Proptosis and ocular motility improved, yet globe displacement remained in 4 patients; all of them had preoperative bony excavation on CT scan (Figures 3(a) and 3(b)), while two patients developed enophthalmos (Figures 3(c) and 3(d)).

## 4. Discussion

Orbital epidermoid cysts are usually mentioned with dermoid cysts although they form a separate and rare pathological entity where few reports are present in literature. These lesions have a wide variety of clinical and radiological presentations and they should be considered in the differential diagnosis of orbital cystic lesions. They usually warrant surgical intervention and should be totally removed as they tend to recur with lipogranuloma formation as well as the remote possibility of malignant transformation [9].

These lesions are usually painless with slow growth over time. Three patients in the current study reported chronic pain, yet there were no signs of orbital inflammation. Ruszkowski et al. suggested that deeply seated dermoid could be associated with pain due to pressure and/or stretch of a related sensory nerve [10].

Clinical presentation varied according to the site and size of these lesions. Lid swelling and presence of a mass were the main findings. Only one patient had an S-shaped deformity due to the location of the cyst behind the lacrimal gland pushing it. Mild proptosis compared to the lesion size was present in 5 patients (29.4%) who were more concerned about ocular asymmetry secondary to globe displacement and lid fullness. The fact of their presence early in life can explain this sign as the bony orbit expands with increased volume in a way similar to what happens with deeply seated dermoids [8].

None of the included patients suffered from diplopia in primary gaze even with globe displacement. It was presumed that ocular adaptation had occurred with anomalous retinal correspondence being of a subtle chronic nature since early childhood. None of the reported cases showed primary presentation of orbital inflammation or fistula formation as has been reported with deeply seated dermoids [7]. However, one case presented with chronic abscess with foul odour content during surgical removal and that was a recurrent cyst for the third time.

Orbital CT helps in the assessment of such lesions and in delineating related bony changes where the lesion density depends on its fat content. Most of the cases included in this study showed hypodense appearance with translucent center, yet the heterogeneous appearance was mostly observed in recurrent cases especially the one associated with chronic abscess.

Arana and colleagues reviewed 37 cases of intradiploic epidermoid cysts in different body sites and described their radiological appearances. They had sclerotic borders on plain X-ray films borders, involvement of both diploic tables, and a density similar to brain parenchyma on CT scanning [2] yet similar to dermoid cysts on MRI [11].

Intradiploic pattern was found in four of the included patients with superior and superotemporal sites, sclerotic appearance of the edges on bone window settings, hourglass appearance of the cyst, and globe displacement. Two of these patients had an associated bony defect in the orbital roof with no intracranial extension. Bony defects have been described in dumbbell dermoids on CT imaging, yet intracranial extension was usually detected [2]. The presence of such bone irregularities and residual edges of the diploë can explain the incomplete globe replacement after the surgical removal of the cyst.

Shields and Shields had classified orbital dermoid cysts according to their relation to suture lines into juxtasutural, sutural, and soft tissue cysts. Juxtasutural cyst is not firmly attached to suture while a sutural dermoid is firmly attached to and usually associated with bone erosion [7, 12]. In the current study, epidermoid cysts had a comparable behavior, where 2 cases were related to frontozygomatic suture with hourglass configuration, while the rest of cases were either juxtasutural or confined to soft tissue.

Keene and coworkers stated that epidermoid cysts are more commonly located laterally in the diploë of the skull, fourth ventricle, and cerebellopontine angle in comparison to dermoid cysts that frequently appear near the midline or superotemporal site where they are related to the frontozygomatic suture [1, 11]. Epidermoid cyst position was variable in the current study; however, the larger number of orbital cases including the soft tissue epidermoid cysts was a contributing factor in the results.

These deeply seated lesions should be differentiated from other orbital cysts such as dermoids, mucoceles, hydatid cysts, cysticercosis in addition to lacrimal gland tumors, and thyroid eye disease in adult presentations as well as encephaloceles in children [5, 10]. Lesions visible through the conjunctival fornices should be differentiated from implantation epidermoid cysts secondary to surgery or trauma as well as dacryops of accessory lacrimal glands that are associated with conjunctivitis, especially trachoma [13].

Histopathology is required to diagnose epidermoid cysts where they are usually lined by stratified squamous epithelium and do not contain either skin appendages, in contrast with dermoids, or goblet cells like implantation cysts. Dacryops have a cuboidal epithelium lining surrounded by a layer of spindled myoepithelial cells and chronic inflammatory reaction [13].

Some authors suggested that patients' age and the site of the lesion can be useful clinical clues. Eijpe and coworkers suggested that epidermoid cysts present later in life due to their deep location and longer latent period as a result of their intradiploic origin in contrast with dermoid cysts that are more common in childhood or young adolescence [2]. The mean age of included patients in the current study was 16.3 years ± 10.54 and the findings concerning intradiploic cysts were comparable, yet this study included other types that showed various age onsets.

Surgical excision is recommended to provide definitive histological diagnosis, correct proptosis, prevent further destruction of related structures, and reduce the potential for malignant transformation [1, 2, 4]. A multidisciplinary approach using transcranial or a temporal skull base approaches was suggested for large intradiploic cysts and cysts located along the orbital roof and temporal fossa [14]. In all situations, the epithelial lining should be completely removed to avoid recurrence.

In the current series, anterior orbitotomy was successfully used in all cases with different incision placements according to site, size, and related changes. None of the combined approaches were required. It is to be noted that these cysts may get opened during surgery; hence, copious irrigation is required and the walls should be traced for complete excision. Only two patients with intradiploic cysts had adherent cyst wall to bone where scraping, irrigation, and cauterization were used to destroy residual cells.

No recurrence was reported during the follow-up period, yet two patients developed postoperative enophthalmos. Both of them were recurrent cases and enophthalmos could be attributed to pressure atrophy and fat necrosis from their prolonged presence, enlarged orbit that was masked by the mass presence, or posttraumatic fat necrosis secondary to surgical manipulations.

In conclusion, deep orbital epidermoid cysts are a separate entity that can behave like deep orbital dermoids; however, they usually present at a relatively older age. They can be associated with increased orbital volume but not necessarily related to bony sutures. Putting clinical and radiological clues in mind can help in planning and give an idea about prognosis. Nevertheless, no effort should be spared to ensure complete excision of such lesions to avoid recurrence.

## Figures and Tables

**Table 1:** Frequency of complaints in patients' own words and clinical signs where a mass with lid swelling is detected in all cases.

Signs and symptoms	Number of patients	%
Complaint		
Lid swelling/fullness	13	76.47%
Visible mass	11	64.7%
Ocular dissimilarity	7	41.17%
Chronic pain	3	17.64%
Ocular protrusion	1	5.88%
Clinical sign		
Lid swelling/fullness	17	100
Associated with S-shaped deformity	1	5.88%
Mass		
Palpable	17	100%
Visible	13	76.4%
With conjunctival part	9	52.94%
Globe displacement	6	35.29%
Proptosis	5	29.4%
Limitation of ocular motility	5	29.4%
Scar of previous surgery		
Skin	2	11.76%
Conjunctival	2	
Diplopia in primary gaze	—	—

**Table 2:** Frequency of various orbital findings in CT scans. Hypodense appears grayish while radiolucent appears black.

CT findings	Number of patients	%
Mass		
Homogenous hypodense	10	58.82%
Homogenous radiolucent	3	17.64%
Heterogenous/irregular borders	4	23.52%
Total	**17 **	**100%**
Site		
Superior/superonasal	9	52.94%
Superotemporal	4	23.53%
Inferior	4	23.53%
Globe displacement	5	29.4%
Increased orbital volume	7	41.17%
Bony excavation	4	23.53%
Bony defect	2	11.76%
